# Emergence of Functional Specificity in Balanced Networks with Synaptic Plasticity

**DOI:** 10.1371/journal.pcbi.1004307

**Published:** 2015-06-19

**Authors:** Sadra Sadeh, Claudia Clopath, Stefan Rotter

**Affiliations:** 1 Bernstein Center Freiburg & Faculty of Biology, University of Freiburg, Freiburg im Breisgau, Germany; 2 Bioengineering Department, Imperial College London, London, United Kingdom; École Normale Supérieure, College de France, CNRS, FRANCE

## Abstract

In rodent visual cortex, synaptic connections between orientation-selective neurons are unspecific at the time of eye opening, and become to some degree functionally specific only later during development. An explanation for this two-stage process was proposed in terms of Hebbian plasticity based on visual experience that would eventually enhance connections between neurons with similar response features. For this to work, however, two conditions must be satisfied: First, orientation selective neuronal responses must exist before specific recurrent synaptic connections can be established. Second, Hebbian learning must be compatible with the recurrent network dynamics contributing to orientation selectivity, and the resulting specific connectivity must remain stable for unspecific background activity. Previous studies have mainly focused on very simple models, where the receptive fields of neurons were essentially determined by feedforward mechanisms, and where the recurrent network was small, lacking the complex recurrent dynamics of large-scale networks of excitatory and inhibitory neurons. Here we studied the emergence of functionally specific connectivity in large-scale recurrent networks with synaptic plasticity. Our results show that balanced random networks, which already exhibit highly selective responses at eye opening, can develop feature-specific connectivity if appropriate rules of synaptic plasticity are invoked within and between excitatory and inhibitory populations. If these conditions are met, the initial orientation selectivity guides the process of Hebbian learning and, as a result, functionally specific and a surplus of bidirectional connections emerge. Our results thus demonstrate the cooperation of synaptic plasticity and recurrent dynamics in large-scale functional networks with realistic receptive fields, highlight the role of inhibition as a critical element in this process, and paves the road for further computational studies of sensory processing in neocortical network models equipped with synaptic plasticity.

## Author Summary

In primary visual cortex of mammals, neurons are selective to the orientation of contrast edges. In some species, as cats and monkeys, neurons preferring similar orientations are adjacent on the cortical surface, leading to smooth orientation maps. In rodents, in contrast, such spatial orientation maps do not exist, and neurons of different specificities are mixed in a salt-and-pepper fashion. During development, however, a “functional” map of orientation selectivity emerges, where connections between neurons of similar preferred orientations are selectively enhanced. Here we show how such feature-specific connectivity can arise in realistic neocortical networks of excitatory and inhibitory neurons. Our results demonstrate how recurrent dynamics can work in cooperation with synaptic plasticity to form networks where neurons preferring similar stimulus features connect more strongly together. Such networks, in turn, are known to enhance the specificity of neuronal responses to a stimulus. Our study thus reveals how self-organizing connectivity in neuronal networks enable them to achieve new or enhanced functions, and it underlines the essential role of recurrent inhibition and plasticity in this process.

## Introduction

Although lacking an orderly map of orientation selectivity (OS) [1–3], an increased connectivity between neurons with similar preferred orientations (POs) has been reported in the visual cortex of adult mice [4–7]. However, such specific connectivity is conspicuously lacking immediately after eye opening [6], suggesting that it might be a result of experience-dependent plasticity during development. Simulations have indeed corroborated that synaptic plasticity can lead to the formation of functionally specific networks [6].

The insight into the neuronal mechanisms that can be derived from those numerical studies is, however, still somewhat limited: First, the results were reported only for very small ensembles of neurons that lacked the rich recurrent dynamics of realistic cortical networks. Second, the receptive fields and OS properties were mainly driven by feedforward inputs, and the effect of large recurrent network dynamics in shaping orientation selective responses of neurons was not taken into consideration. It remains, therefore, unclear if feature-specific connectivity can at all emerge in more realistic neuronal networks, and whether the recurrent dynamics possibly compromises the stability of the learned weights.

Here we study this scenario in large-scale recurrent networks of excitatory and inhibitory neurons. These networks are known for their rich and biologically realistic repertoire of the dynamics they can exhibit [8, 9]. As a result, it is not clear if the connectivity structure observed experimentally in adult animals [6] can also be obtained in simulations under these more realistic conditions. Collective dynamics of a strongly recurrent network of excitatory and inhibitory neurons may enhance, but could also impede Hebbian learning and self-organization [10, 11]. The dynamics of large plastic networks is difficult to characterize in general [10, 12–22], and there is conflicting evidence whether functional subnetworks can emerge in them under biological conditions. It remains, therefore, unclear if functionally specific connectivity can develop in such networks.

Even more challenging is the question whether specific connectivity can be obtained in balanced networks of spiking neurons with realistic receptive fields. It has been recently demonstrated that balanced random networks can show highly selective neuronal responses despite receiving only weakly tuned inputs [23–25]. Here we report that both highly selective neuronal responses and functionally specific connectivity emerge simultaneously in balanced random networks with synaptic plasticity of excitatory-excitatory and excitatory-inhibitory recurrent connections. In addition, the resulting connection specificity is sensitive to the statistics of the stimuli, and an over-representation of one particular orientation leads to an over-representation of functionally specific weights among those neurons that prefer this orientation. Interestingly, the emerging functional subnetworks remain stable for spontaneously active (non-stimulated) networks, and the activity of the network in absence of a visual stimulus does not substantially change the learned weights.

## Results

**Fig 1:**
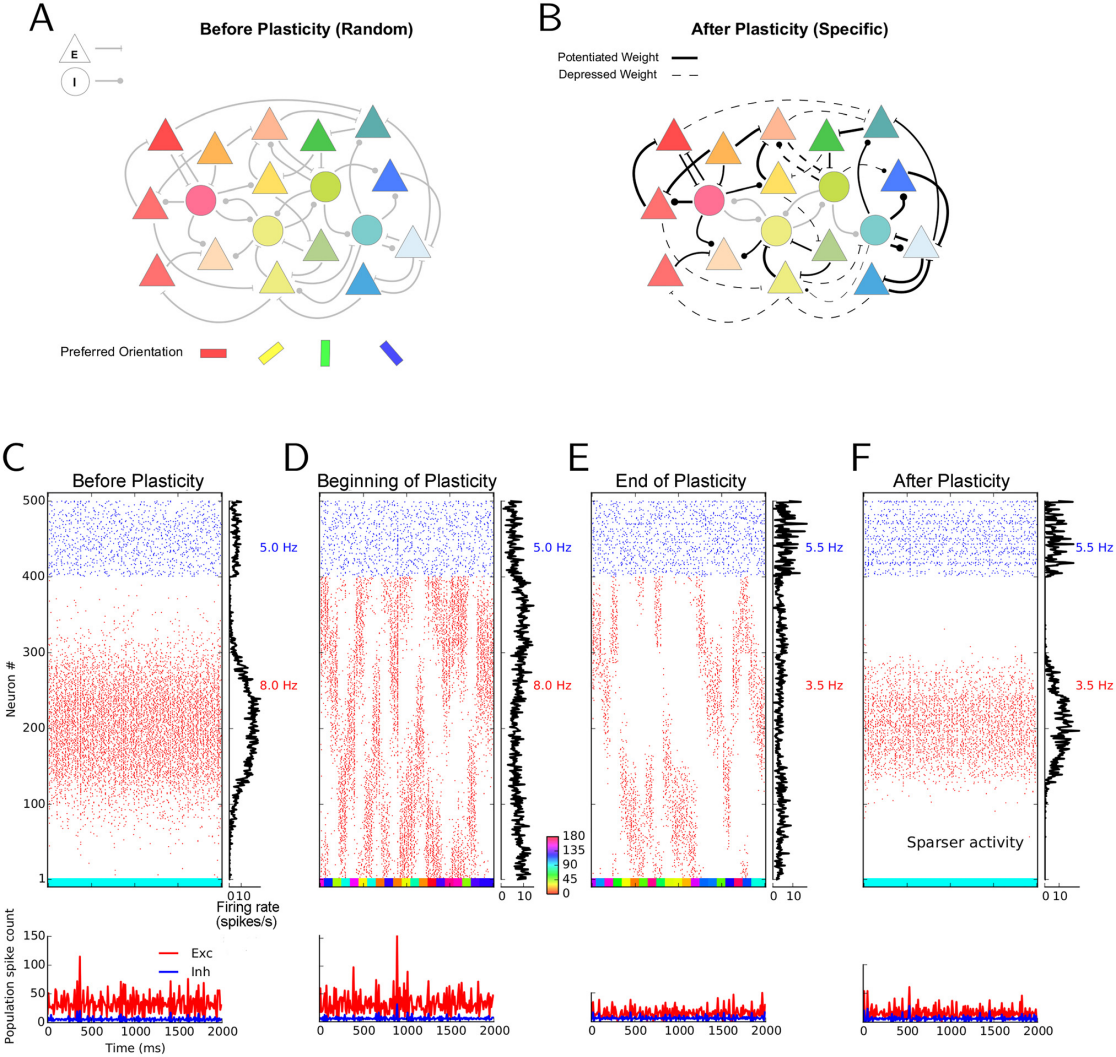
Simulating the effect of synaptic plasticity in balanced random networks. (**A**) In random networks of excitatory (E; triangles) and inhibitory (I; circles) neurons, synaptic connections are established disregarding the stimulus selectivities (preferred orientation) of pre- and post-synaptic neurons. (**B**) In specific networks, synapses between neurons of similar preferred orientations are stronger, while dissimilar feature selectivity of pre- and post-synaptic neurons imply weaker synapses between them. (**C–F**) Stimulus-induced response of a network before, during and after learning. The middle of each panel shows the raster plot of two seconds of stimulation. Spikes of excitatory and inhibitory neurons are displayed in red and blue, respectively. Within each population, neurons are sorted according to the preferred orientations of their weakly tuned inputs. Average firing rates of individual neurons during the period of stimulation are shown in the histogram on the right. The average firing rate of each subpopulation is indicated to the right of it, in the corresponding color. The lower panel depicts the time-resolved histograms of population activity for excitatory (red) and inhibitory (blue) neurons, respectively. Population spike counts are extracted from bins of size 10 ms. The colored bar at the bottom of the main panel shows the sequence of stimulus orientations applied during the simulation (color code is indicated between panels (D) and (E)). For the simulations before (C) and after (F) learning, the initial or final weights are frozen, respectively, and network activity is simulated with static weights in response to a stimulus of orientation 90°. During learning, a network with plastic synapses is stimulated with 40 batches of oriented bar stimuli. Each batch consists of a random sequence of 20 different stimulus orientations, each stimulus lasting for 100 ms. Therefore, the “visual experience” lasts 20 × 20 × 0.1 s = 40 s in total. The responses to the first and the last batch are shown in (D) and (E), respectively.

Our point of departure is a recurrent network of excitatory and inhibitory neurons, with sparse random connectivity (Fig 1A). Both the connection probability and the synaptic weights are taken to be independent of the orientation preference of pre- or post-synaptic neurons. We now ask the question whether such a network with plastic recurrent synapses can, through visual experience, develop specific connectivity (Fig 1B), where neurons with similar preferred orientations are more likely and/or more strongly connected than others. To simplify our simulations and analyses, we admit only changes in the strength of synapses here. Structural plasticity is not considered here and, hence, the multiplicity of connections is fixed in the networks considered (see Materials and Methods). Feedforward plasticity is also not considered, and we assume a fixed weight for the feedforward connections in all our simulations (see Discussion).

### Activity-dependent modification of network responses

In agreement with experiments in mice demonstrating that neurons show orientation selective responses already at eye opening [6], neurons in our networks exhibit selective responses even before synaptic plasticity is turned on in the network, and despite receiving only weakly tuned inputs [6, 23–25]. This is demonstrated here with the example of network activity in response to a stimulus orientation of 90° (Fig 1C).

We then studied the consequences of turning on synaptic plasticity in our networks. We implemented a voltage-based spike-timing dependent plasticity (STDP) rule [26] for excitatory-to-excitatory (E → E), excitatory-to-inhibitory (E → I), and inhibitory-to-excitatory (I → E) connections, while keeping inhibitory-to-inhibitory (I → I) connections non-plastic (see Materials and Methods). This particular learning rule has been shown to faithfully reproduce a broad range of experimental results on pair-based spike-timing-dependent plasticity, STDP [27, 28], voltage-clamp experiments [29, 30]), frequency dependency [31], pair and triplet experiments [32], and triplet and quadruplet experiments [33] (see [34] for details). We then stimulated the plastic network with a random sequence of different orientations, reflecting some visual experience of this circuit of total duration 80 seconds. The response of the network at the beginning and at the end of the learning period are depicted in Fig 1D and 1E, respectively. The raster plots and histograms of population activity show that plasticity-induced changes cause sparser activity in the network in the excitatory population. Individual neurons are selective before and after learning, but after learning they respond with a lower number of spikes, and to a reduced range of stimulus orientations. This is most evident when we freeze the final weights after learning and stimulate the network with exactly the same input as before learning (compare Fig 1C and 1F). The overall response of the network activity (as reflected by the “network tuning curve”) is still selective, but fewer spikes are emitted, and a smaller set of neurons is now active.

### Individual tuning curves before and after experience-dependent plasticity

**Fig 2:**
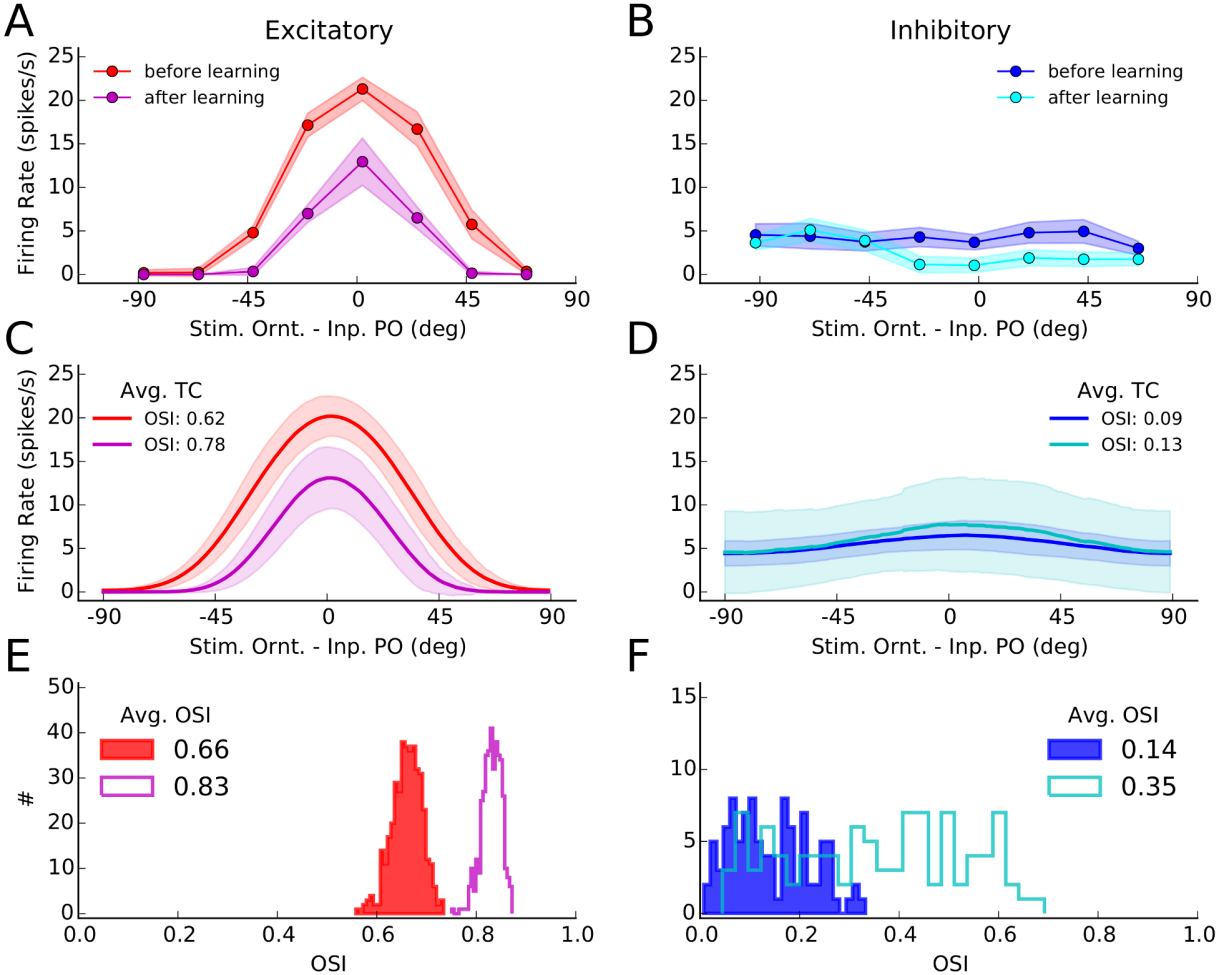
Orientation selectivity before and after learning. **(A)** Tuning curves of a sample excitatory neuron from the network before and after learning. The mean firing rate of a neuron for different stimulus orientations was extracted from simulations of network activity for two seconds, as explained for Fig 1C and 1F. This was repeated 10 times for each orientation, and the mean and standard deviation over trials of the firing rates are depicted by solid lines and corresponding shadings, respectively. **(B)** Same as (A) for a sample inhibitory neuron. **(C)** All tuning curves of excitatory neurons, similar to the tuning curve in (A), were aligned to the preferred orientation of the input, and the average tuning curves (over 180 bins) before and after learning were computed from them. The solid line indicates the mean value in each bin, and the shading is ± one standard deviation. The orientation selectivity index indicated in the plot (OSI, see Materials and Methods) was computed from the average tuning curves, respectively. **(D)** Same as (C) for the inhibitory population. **(E)** The OSI was computed for each individual tuning curve, and its distribution for the entire excitatory population is shown for a network before (solid) and after (outlined) learning. The average OSI in the network is indicated for each case. **(F)** Same as (E), for the inhibitory population.

Changes in neuronal responses as a result of learning can be further illustrated by individual tuning curves (Fig 2). We obtained such tuning curves by stimulating the network before and after learning (Fig 1C and 1F, respectively), for 8 different stimulus orientations. Tuning curves were extracted from the average firing rates of neurons in response to each orientation. Shown are a sample excitatory and a sample inhibitory neuron in Fig 2A and 2B, respectively, and the average tuning curves of the respective subpopulations in Fig 2C and 2D, respectively. Such average tuning curves are obtained from averaging over individual tuning curves each aligned to its respective input preferred orientation.

Overall, tuning curves show a slight enhancement of selectivity, as quantified by the orientation selectivity index, OSI (see Materials and Methods). The excitatory population responds with less firing rate, consistent with our observation above that plasticity leads to sparser and more selective network responses in this case. Enhancement of orientation selectivity, for individual cells, also shows up in the distribution of OSI of individual tuning curves before and after learning (Fig 2D and 2E, for excitatory and inhibitory populations, respectively).

This enhancement of tuning in excitatory neurons is consistent with the results reported by Ko et al. [6], who demonstrated a slight enhancement of OSI after eye opening (an increase from 0.62 to 0.68, see supplementary Figure 2 therein). Another experimental study [35] has in fact reported a larger (almost two-fold) enhancement of OSI (from ≈ 0.4 to 0.8, see Figure 4B therein) during development. Note, however, that a different measure of orientation selectivity (i.e. (*R*_pref_ − *R*_orth_)/(*R*_pref_ + *R*_orth_), where *R*_pref_ and *R*_orth_ are firing rates at preferred and orthogonal orientations, respectively) were employed in these two experimental studies, as opposed to the “global” measure of orientation selectivity (1−circular variance, see Materials and Methods) that we have used here [36, 37]. A very recent study [38] has in fact computed both measures of orientation selectivity and found a significant enhancement of OSI for both measures.

### Emergence of feature-specific connectivity through learning

**Fig 3:**
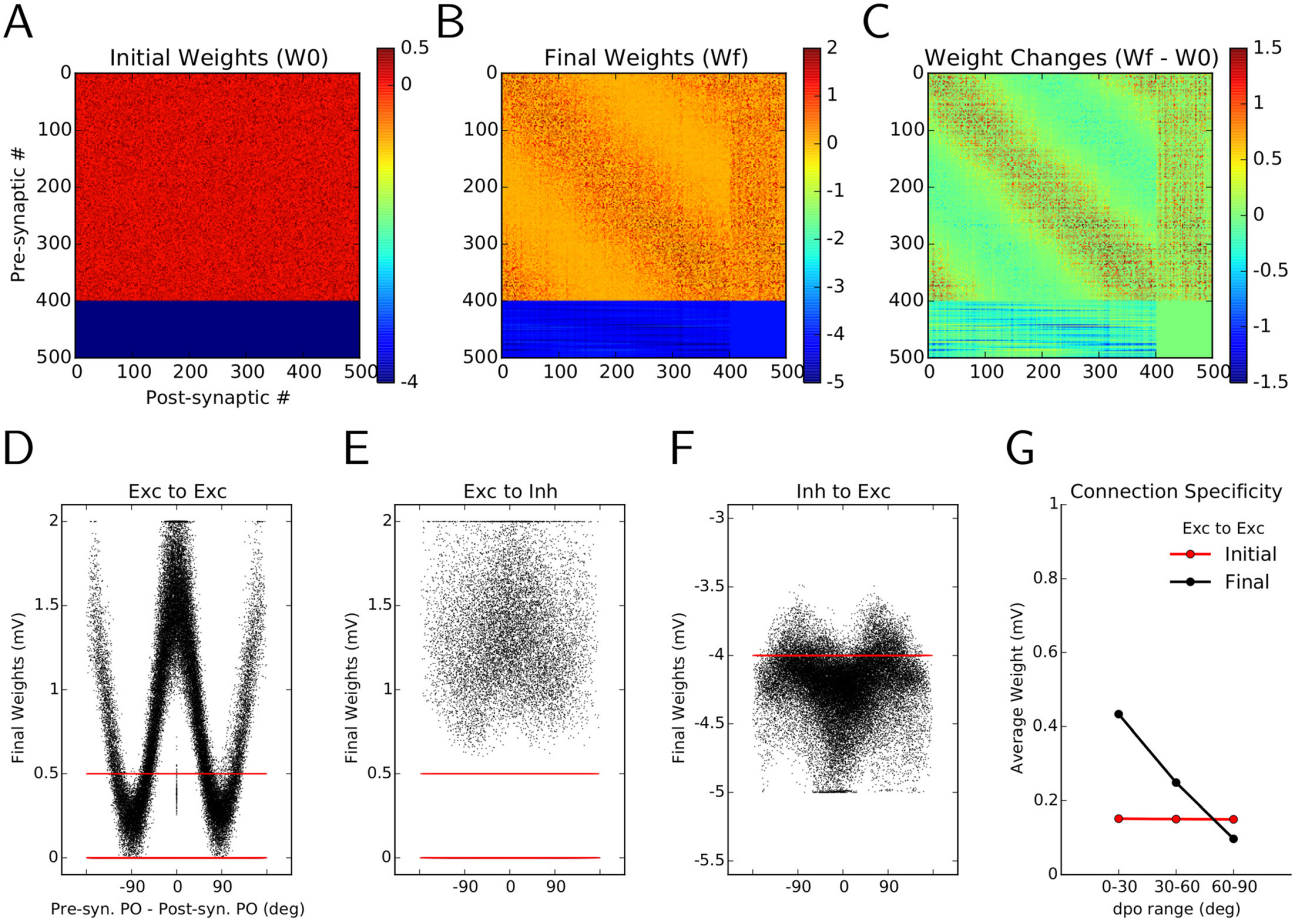
Emergence of specific connectivity as a result of synaptic plasticity. **(A)** Initial connectivity matrix of the random network. Each excitatory neuron is connected to a random sample of 30% of all post-synaptic neurons, with a weight EPSP = 0.5 mV. Inhibitory neurons form synapses of weight IPSP = −4 mV. Neurons are sorted according to their preferred orientations (POs) within each population (1–400: excitatory, 401–500: inhibitory). **(B)** Matrix of synaptic weights in the network, after learning. **(C)** Change in the weights as a result of plasticity and “sensory experience”. The overall weight increase on the diagonal shows the specific potentiation of synapses between pairs of excitatory neurons with similar POs. **(D–F)** The final weights (black) are plotted against the difference in the preferred orientations (dPO) of pre-synaptic and post-synaptic neurons, for E → E(D), E → I (E) and I → E (F) connections, respectively. The initial weights are shown in red, for comparison. **(G)** Mean of the weight distributions are shown for all synapses between neurons with similar (dPO < 30°), indifferent (30° < dPO < 60°) and dissimilar POs (dPO > 60°). The unspecific distribution of initial weights (red) has now changed to an over-emphasis of connections between neurons with similar POs, indicating the emergence of specific connectivity in the network through synaptic plasticity and visual experience.

The synaptic learning rule considered here allows for changes in the weights of connections. Comparing the initial random connectivity matrix (Fig 3A) with the connectivity matrix after learning reveals some interesting structure within its subpopulations (Fig 3B). This pattern becomes more salient when the accumulated changes of weights are plotted directly (Fig 3C). One observes an unspecific potentiation (increase in amplitude) of E → I and (I → E) connections (column numbers and row numbers 401 to 500, respectively). This explains the observed sparser responses in the network as the result of a more intense recruitment of inhibitory neurons.

More noteworthy, however, is the increase in some E → E connections (diagonal band in the matrix (B) and (C)). As neuron indices are sorted according to the preferred orientation of the input to neurons, an increase around the main diagonal indicates a potentiation of connections between neurons with similar preferred orientations. The obvious symmetry of the E → E block implies an over-representation of bidirectional connectivity between these neurons, as was also shown experimentally [6]. This can be quantified by a normalized measure of weighted bidirectional connectivity (WBI_norm_, see Materials and Methods), which has been increased here from 1 (bidirectional connections expected from a random distribution of weights) before plasticity to 1.38 (38% over-representation of bidirectional weights compared to random distribution) after plasticity. As was noted in a previous small network model [6, 26], with an architecture that was mainly driven by feedforward links, this increase in bidirectional connectivity is a consequence of the employed plasticity rule and would also hold for other, similar nonlinear rules [39–42]. Symmetric pair-based STDP models [14, 43], however, would not support bidirectional connectivity.

Fig 3D–3E show the distribution of synaptic weights after learning, in relation to the difference in preferred orientation of pre- and post-synaptic neurons (dPO). The clear tuning of weights for E → E connections (Fig 3D) demonstrates the emergence of specific connections (black) in the network from initial random connections (red). In the case of E → I (Fig 3E) and I → E connections (Fig 3F) the specific enhancement of weights is generally much weaker, reflecting the aforementioned essentially unspecific potentiation of all connections. The reason for this is the weaker tuning of neuronal responses in the inhibitory population (Fig 2A–2D), as was also observed experimentally [4, 44, 45]. In our simulations, this is in turn a result of a weaker tuning of feedforward input to inhibitory neurons (see Materials and Methods).

The sharp tuning of E → E weights as a function of the difference between preferred orientations (dPO) of the pre- and post-synaptic neurons leads to an over-emphasis of connections between neurons with similar preferred orientations. If pre-post pairs are split according to the difference of their preferred orientations to similar (dPO < 30°), indifferent (30° < dPO < 60°) and dissimilar groups (60° < dPO < 90°), a clear trend is now observed (Fig 3G). In contrast to random networks with a flat profile of average connectivity (before learning, red), after the plastic period in the balanced network pairs of excitatory neurons with similar POs increase, and pairs with dissimilar POs decrease the strength of a synapse between them (after learning, black), fully consistent with experiments [5, 6].

In the results presented above, we considered networks with a connection probability of 30% for both E → E and E → I connections. An even higher connection probability for E → I has in fact been reported in an experimental study [4]. We therefore studied how such a difference in connectivity affects our results. We found that all our main results, including emergence of feature-specific connectivity and sparse orientation selective responses after learning, also hold for the alternative connectivity parameters (S1 Fig). Our results are therefore also consistent with a more experimentally constrained network connectivity. Note, however, that matching the parameters of our model networks to real cortical networks might not be straightforward, as relatively small networks of 500 neurons were considered to speed up the simulations, and the exact scaling of the connectivity parameters for larger networks might be different. Our conclusions, however, hold for a range of connectivity parameters as long as the network is inhibition-dominated and operates in a balanced activity regime.

### Balanced activity regime before and after learning

**Fig 4:**
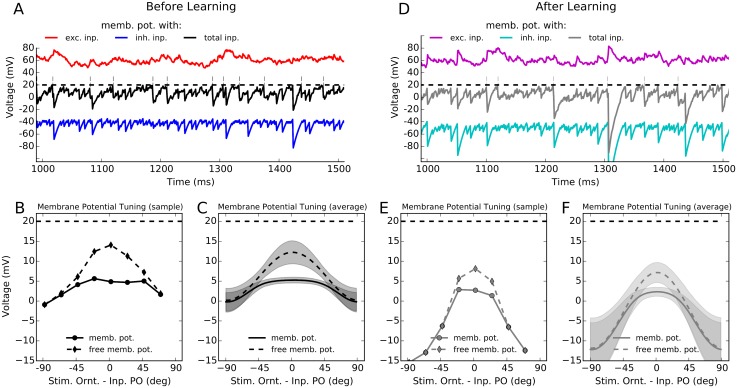
Balanced activity regime before and after learning. **(A)** The membrane potential trace of the same excitatory neuron shown in Fig 2A in response to its preferred orientation is depicted here for 500 ms. The black trace is its actual membrane potential, with the thin vertical lines denoting spikes when the membrane potential crosses the spiking threshold (the dashed line). The red and the blue traces reflect the excitatory and inhibitory components of the free membrane potential (by not allowing the neuron to spike), respectively. **(B)** Temporal average of the membrane potential, ⟨*u*⟩, over two seconds of stimulation is extracted for each stimulus orientation and plotted as a tuning curve. The tuning curve of the free membrane potential, *u*_free_, is also computed, by correcting for the total reset voltage induced as a result of spiking, i.e. *u*_free_ = *u* + *τ*_*m*_ *u*_th_ *r*, where *r* is the mean firing rate of the neuron. **(C)** The tuning curves introduced in (B) for a single cell are now computed for the whole network, by aligning the individual tuning curves and computing the mean and standard deviation of values in each 180 discrete bins (similar to Fig 2C). **(D–F)** Same as (A–C), respectively, for the network after learning.

The balanced change of synaptic weights ensures that balanced regime of activity is maintained in the network also after learning (Fig 4). In either case, a large excitatory input to the neurons is counter-balanced by recurrent inhibition of comparable strength, which keeps individual neurons away from saturation, and prevents runaway activity to occur in the network. This is demonstrated for a sample neuron in response to its preferred orientation before and after learning (Fig 4A and 4D, respectively). A 500 ms trace of the actual membrane potential of the neuron is shown, along with the excitatory and inhibitory components of its (spike free) membrane potential.

As a result of balanced input to the neuron, its membrane potential remains on average below threshold, and spikes are generated by positive-going fluctuations. The tuning curve of the average membrane potential of a sample neuron before and after learning (Fig 4B and 4E, respectively), as well as the average (over network) tuning curves of the membrane potential of all neurons in the network also reveal the same behavior (Fig 4C and 4F). Both networks, before and after learning, operate in the balanced activity regime. The only difference is that the network after learning shows more hyper-polarized membrane potentials (Fig 4F). This is explained by the potentiated inhibition shown in Fig 4D, as compared to the network before learning. This is also fully consistent with sparser and more selective responses of networks after activity-dependent plasticity.

Orientation selectivity that emerges in balanced networks is very pronounced, even if the selectivity of the feedforward input is extremely weak. This is because the untuned component of the input is strongly suppressed by balanced networks [23], as a result of selective attenuation of the common-mode due to dominant recurrent inhibition, while the same mechanism does not affect the tuned component [25]. This processing is fully contrast-invariant: If the tuned component of the input remains the same while only the untuned component increases (leading to a decrease of the relative modulation depth of the input), balanced networks still maintain their output selectivity.

This is exactly the scenario when the typical connectivity in the network, *C* = *ϵN* (corresponding to the average number of connections each neuron receives in the network, see Materials and Methods), increases. In this case, the untuned component scales with *C*, while the modulation scales with $\sqrt{C}$. As a consequence, the relative modulation amplitude of the input tuning (denoted by *μ* in our model, see Materials and Methods), also scales with $1/\sqrt{C}$ [23]. We therefore investigated the response of our networks for different input selectivities in this manner, to see if our networks invariantly respond to more weakly tuned inputs.

**Fig 5:**
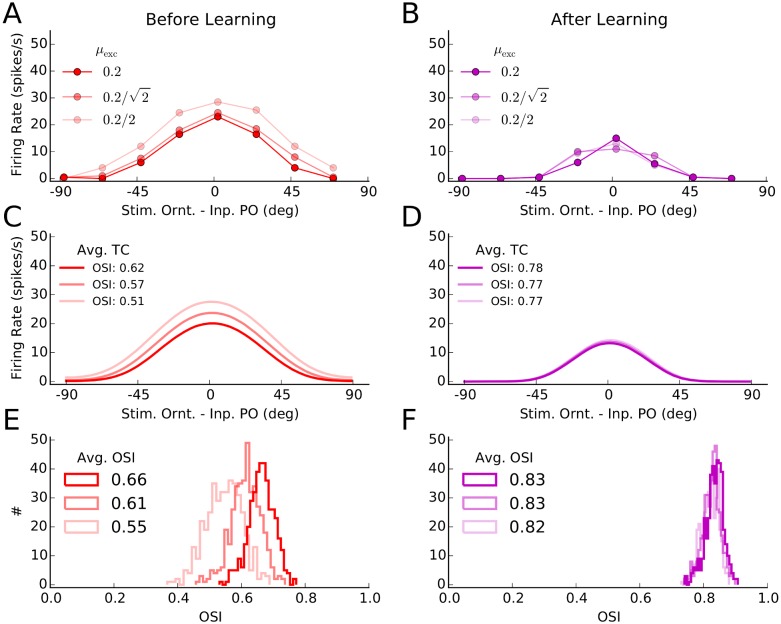
Orientation selectivity of network responses to more weakly tuned inputs. **(A)** Simulations of the network in Fig 2 (before learning) are repeated with the following changes in the parameters: *s*_*b*_ (see Eq 2 in Materials and Methods) is scaled by a connectivity factor (*C* = 1, 2, 4), which emulates an effective increase in connectivity. The relative modulation of the feedforward input to excitatory neurons, *μ*_exc_ (Eq 2), is reduced by a factor $1/\sqrt{C}$. Finally, to keep the absolute size of modulation the same, feedforward synaptic strength is scaled by a factor $1/\sqrt{C}$. The output tuning curve of the sample neuron in Fig 2A for the three different values of *C* is then plotted. **(B)** Same as (A) for the network after learning. **(C)** Average tuning curves of all the cells in the network (extracted similarly to Fig 2C) are plotted for different values of *C*, along with the OSI computed for each average tuning curve. **(D)** Same as (C) for the network after learning. **(E, F)** Distribution of OSI of individual tuning curves (similar to Fig 2E and 2F) for the networks before and after learning, respectively.

To study this situation, we decreased the relative modulation of input to excitatory neurons, *μ*_exc_, in our networks, while scaling the strength of input such that the absolute modulation amplitude in the input remained the same (Fig 5). The tuning curves of a sample cell are, in fact, almost the same before and after learning (Fig 5A and 5B). Output selectivity is maintained despite a two-fold decrease in input selectivity.

This effect can be better seen, on the level of the entire population, by plotting the average tuning curve of neurons in the network (Fig 5C and 5D), and the distribution of OSI of individual tuning curves (Fig 5E and 5F). Robust output selectivity is again observed for both networks, before and after learning. However, although there are some subtle differences among different tuning curves before learning, the network after learning shows completely identical responses to different inputs. Plasticity of excitatory and inhibitory weights, therefore, also extends the invariance of balanced network responses to more weakly tuned inputs.

### Dynamics and stability of learning in the network

**Fig 6:**
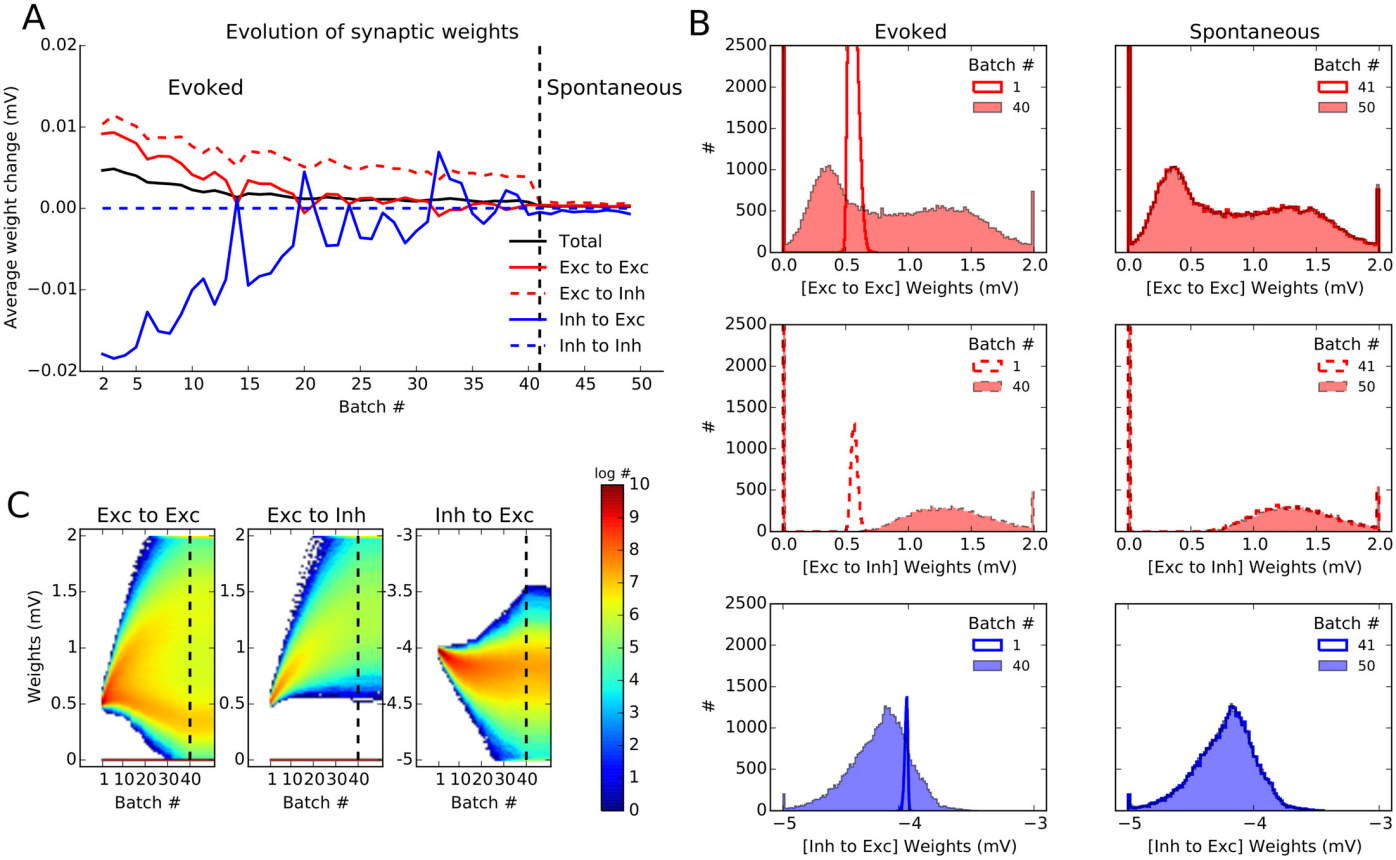
Dynamics and stability of learned weights. **(A)** Evolution of synaptic weights in the network during plasticity. After each batch of learning, weights are frozen and compared to the weights in the previous batch. The difference in the weights is averaged over populations, for all synapses, respectively. On average, weight changes decrease over batches, indicating the convergence of the learning process. The weights established under evoked activity are also stable for spontaneous activity, in absence of any specific stimulation (batch 41 and later, after the vertical dashed line). Here, the plasticity period is continued for extra 10 batches, where neurons are only receiving an untuned background input (with an input rate of *s*_*b*_/2 and *μ* = 0, see Materials and Methods). **(B)** Distribution of weights are shown separately for E → E, E → I and I → E weights, for evoked and spontaneous states, respectively. In each case, two distributions are shown, one sampling the beginning and one sampling the end of the corresponding learning phase. **(C)** Distribution of weights extracted at the end of each trial batch (every 2 s) is plotted for all plastic connections in a pseudo-color map. Note the logarithmic scale of the color code.

Next we looked at the dynamics of plasticity in our networks (Fig 6). Following the time evolution of the synaptic weights (averaged over synapses and average per batch) indicates that the connection weights converge smoothly to their equilibrium values during the period of evoked activity of the network (Fig 6A). Importantly, the learned weights remains stable during spontaneous activity: We continued the stimulation with an input that lacks feedforward tuning and has a weaker baseline intensity (reflecting the fact that in the spontaneous state the feedforward input and hence the untuned input induced by it would be weaker). The results showed that the weights learned in the stimulus-induced state now remain stable during untuned input, when the network exhibits only sparse spontaneous activity (Fig 6A). We can therefore conclude that the learned weights generally remain stable even when the corresponding visual stimuli are not present, and govern the evoked responses in future stimulations. Note that this phenomenon could not have been studied in the small network model mentioned before [6], as is the networks studied there were mainly driven by feedforward, stimulus-induced input, and were lacking the realistic recurrent dynamics of large-scale networks we study here.

The convergence of synaptic weights we observed in Fig 6A was for the average weights over synapses. To obtain more insight on the evolution of synaptic weights on the systems level, it is important to also look at the distribution of weights, rather than only on their mean values (Fig 6B and 6C). The distribution of weights before and after learning in the evoked state (first column) and during spontaneous activity (second column) is plotted for E → E, E → I and I → E connections, respectively (Fig 6B). A more complete account of the evolution of weight distributions can be obtained by inspecting the changes in weights after each batch of learning (Fig 6C). Both analyses corroborate the previous results on the convergence of the mean weights, and the stability of weights under spontaneous activity.

However, in the case of E → E weights, as opposed to other connections, a bimodal weight distribution emerges out of the initial distribution of weights. This is due to the fact that the learning rule considered here is hyper-competitive. Therefore the weights are typically pushed either to very high or to very low values. Similar bimodal distributions have previously been reported in several other studies [14, 46, 47]. We did not observe such a bimodal distribution when we chose the initial (non-zero) excitatory weights from a normal distribution about *J*_exc_ (S2 Fig) (for a similar observation, see [14]).

In both cases (Fig 6 and S2 Fig), however, imposing hard bounds on E → E weights seems necessary: in Fig 6C, the emergent bimodal distribution is not stable and smaller and larger weights are still changing (decreasing and increasing, respectively). As a result, if learning is continued for a longer time, smaller weights converge to zero and larger weights converge to the maximum weight imposed on the connections. Such a bimodal distribution at *w*_min_ and *w*_max_ is already visible in S2 Fig.

**Fig 7:**
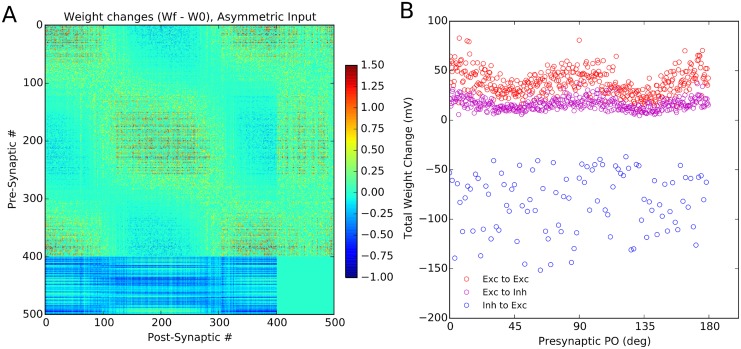
Stimulus statistics is reflected in the learned weights. **(A)** Sensitivity of the final weights to an over-representation of certain stimulus orientations. When the stimulation is repeated with cardinal orientations being more frequent than others (0° and 90° stimuli represent 50% of all stimuli), the final weights also reflect this over-representation of the corresponding subnetworks. **(B)** Total weight change of each presynaptic neuron is plotted vs. its initial preferred orientation (PO). Specifically for the excitatory population, neurons with POs close to 0° and 90° show the highest increase in their synaptic weights.

To further study the issue of dynamics of learning in our networks, we can also investigate whether the network is sensitive to the statistics of stimulation (Fig 7). To test that, we presented cardinal orientations more frequently to the network such that half of stimuli corresponded to either 0° or 90°. We observed that, as a result, the subnetworks corresponding to these orientations became over-represented in the network, as reflected by its connectivity matrix (Fig 7A). Although neurons with similar preferred orientations were still connected more strongly, the enhancement of connectivity is more pronounced for subnetworks preferring over-represented orientations, here corresponding to the cardinal orientations (Fig 7B). Thus, the Hebbian learning leads to the emergence of feature-specific connectivity, while it remains sensitive to the statistics of stimulation.

### Joint emergence of feature selectivity and selective connectivity

Our results so far revealed a critical role of inhibition for the initial establishment of orientation selectivity and the ensuing emergence of feature-specific connectivity. The former was governed by the network dynamics alone, and specifically was a result of inhibition-dominance in our networks. For the latter plasticity of certain recurrent synaptic connections was needed. Next we asked the question what would happen if the initial selectivity was not as strong as we had assumed so far in our simulations underlying Figs 1–7.

**Fig 8:**
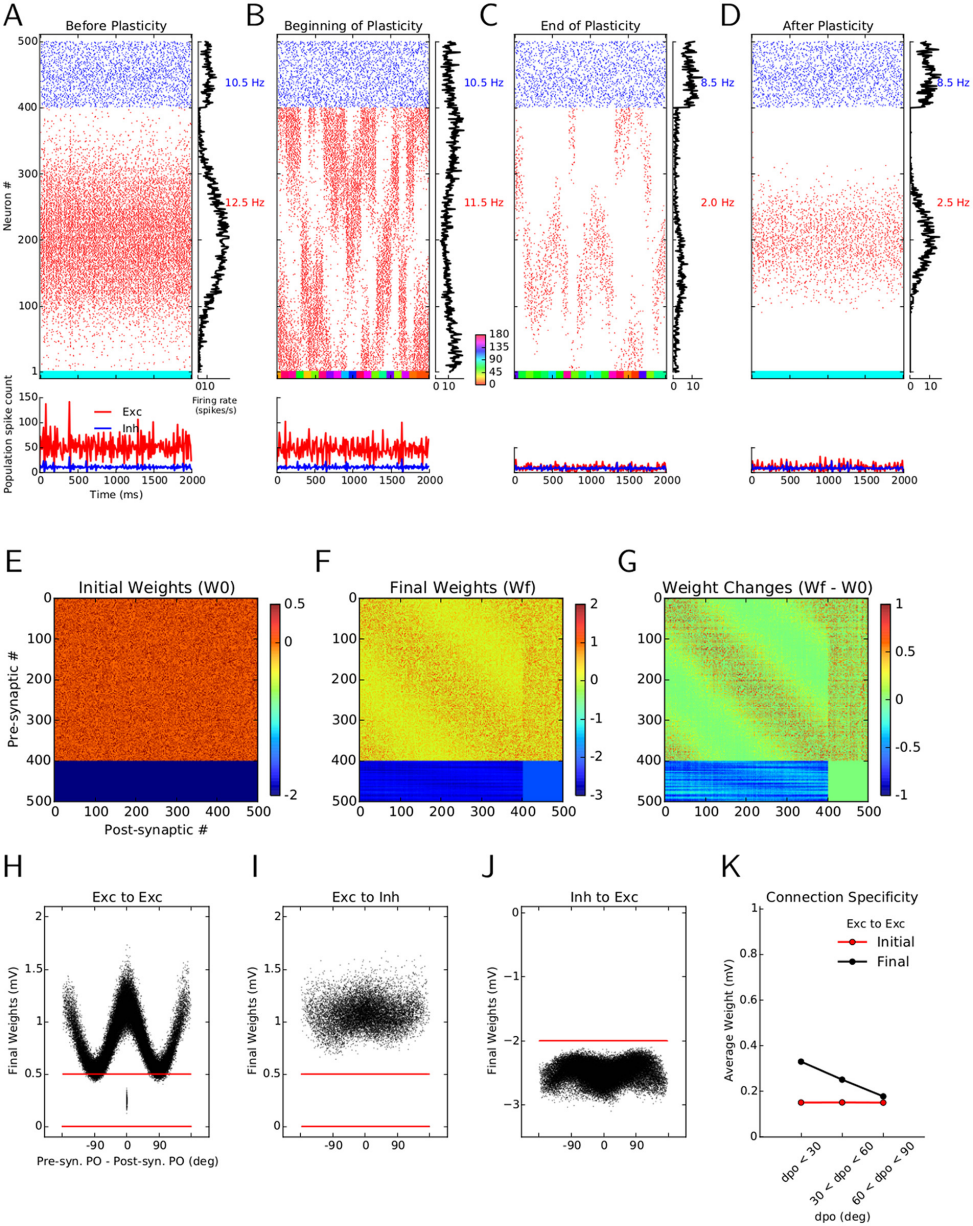
Emergence of feature selectivity and feature-specific connectivity. The format of (A–D) is identical to Fig 2, (C–F), and (E–K) identical to Fig 3, respectively, for a network with initial random connectivity and strongly reduced inhibition dominance, *g*. Instead of *g* = 8, the inhibition-dominance ratio is now decreased to *g* = 4 for the network “before plasticity”. As before, the learning was run for 20 batches.

To answer this question, we first decreased the dominance of inhibition by a factor of 2 (from *g* = 8 to *g* = 4). This lead to a network that was not any more dominated by inhibition, rather excitation and inhibition were of equal strength. Under these conditions, the output selectivity of the network response was not as strong as before (Fig 8A), and the response was generally less sparse. During learning, however, the responses became gradually sparser and highly selective (Fig 8B and 8C), and eventually a degree of selectivity was obtained comparable to the case *g* = 8 considered before (Fig 8D). As a result of synaptic learning in the network, inhibition became more dominant, which ensured highly selective output responses. Notably, feature-specific connectivity was found to result from this procedure at the same time (Fig 8E–8K). Thus, enhancement of feature selectivity and emergence of feature-specific connectivity jointly result from plasticity in randomly connected recurrent networks, even if they are not, or only weakly dominated by inhibition.

**Fig 9:**
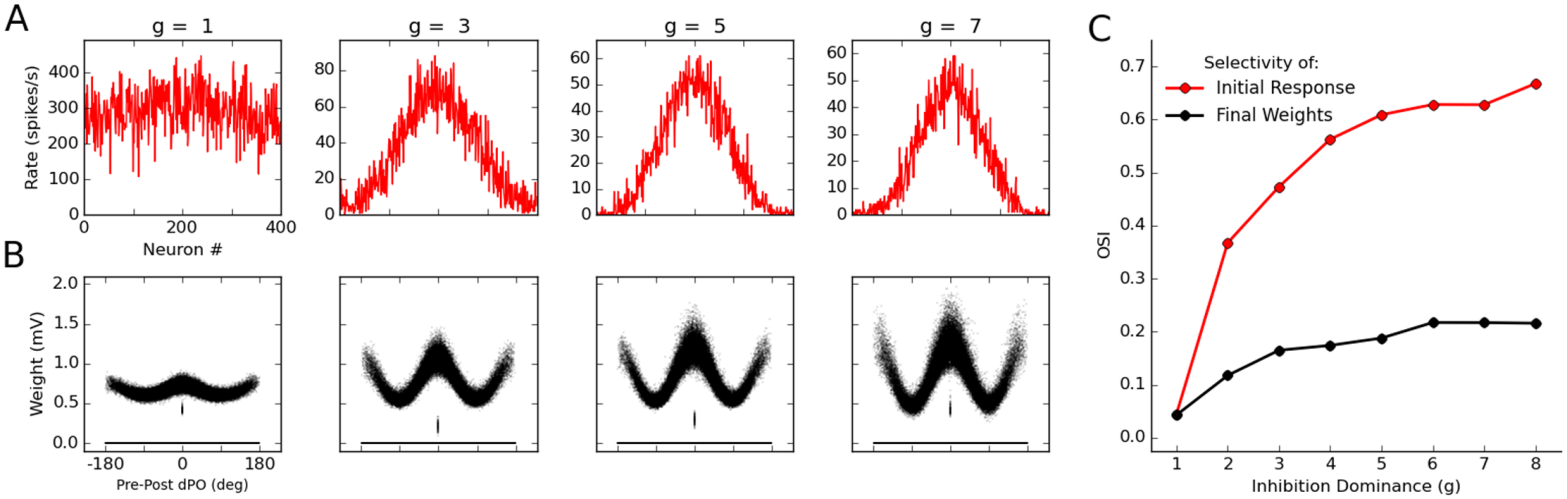
Relation between orientation selectivity and feature-specific connectivity induced by learning. **(A)** Initial (before learning) response selectivity for networks with different amounts of inhibition dominance, *g*. **(B)** Each network is simulated for 20 batches of learning, and the final selectivity of E → E weights is illustrated by plotting the weights vs. the difference in the PO of pre- and post-synaptic neurons (pre-post dPO). **(C)** The selectivity of the initial network tuning curve (A) and the final weight tuning (B) is quantified by computing their OSI (see Materials and Methods) for each value of *g*. The more selective the initial orientation selectivity is, the more feature-specificity is observed in the final connectivity.

To study how feature-specific connectivity is related to initial orientation selectivity in our networks, we systematically changed the inhibition dominance ratio, *g*, and measured the selectivity of E → E weights after learning in each network (Fig 9). The more dominant inhibition was in the beginning, the more selective were the initial network tuning curves (Fig 9A). In all networks, some degree of feature-specific connectivity emerged after learning (Fig 9B). However, the post-learning connections were more specific in networks with initially more selective responses, as revealed by quantifying the selectivity of initial network responses and specificity of final network connections after a similar period of learning (Fig 9C).

### Robustness of the results

The above result shows that the emergence of feature-specific connectivity is robust with regard to the most important parameter of network dynamics, namely inhibition-dominance. We have shown before that the emergence of orientation selectivity is in turn robust to other parameters of network connectivity (specifically, spatial extent of excitation and inhibition in locally connected networks) [48], network dynamics (specifically, operating in asynchronous vs. synchronous or mean-driven vs. fluctuation-driven regimes of activity) and single cell properties (specifically, linear or non-linear neuronal transfer functions) [49]. We will therefore only focus on the parameters of plasticity for the remainder of the paper and test whether our results are also robust to changes in these parameters.

**Fig 10:**
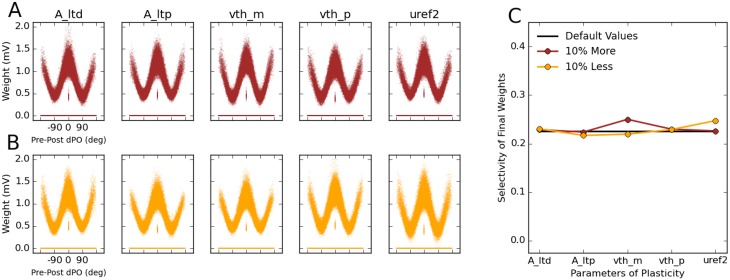
Robustness of the results to changes in the plasticity parameters. **(A, B)** The robustness of the results in the default configuration of the network is studied by decreasing (A) or increasing (B) 5 parameters of plasticity by 10%. The parameters are *A*_LTD_, *A*_LTP_, *θ*_−_, *θ*_+_ and $u_{ref}^{2}$, respectively. The learning phase for each network is again organized in 20 batches. **(C)** Selectivity of the final weights is quantified (blue and red; similar to Fig 9) and compared with the selectivity in the default case (black).

To do this, we changed (either decreased or increased) the major parameters of plasticity in our networks (see Materials and Methods) by 10% of their respective values, and ran the simulations again (Fig 10). In all cases, feature-specific connectivity still emerges in the network, as illustrated by the tuning curve of connection weights as a function of the difference between preferred orientations of pre- and post-synaptic neurons, dPO (Fig 10A). This is further quantified by computing a selectivity index of this tuning curve, which reveals similar values as for simulations with the default values for all plasticity parameters (Fig 10B). We conclude that the emergence of feature-specific connectivity is a robust property of our networks, as it is essentially invariant to wide changes in network parameters.

We also checked specifically whether our results depend on the learning rates of excitatory and inhibitory weights, and found that these are again not crucial for our results (S3 and S4 Figs). We increased the strength of synaptic potentiation (*A*_LTP_; see Materials and Methods) by 20% for either excitatory (S3 Fig) or inhibitory (S4 Fig) synapses, while keeping *A*_LTP_ fixed for the other type of synapses. This lead to similar results, and we found that the emergence of feature-specific connectivity in our networks is not compromised by different learning rates. The only difference was a slightly increased or decreased feature-specificity of connections when the learning rate was larger for excitatory and inhibitory synapses, respectively (S3 and S4 Figs, respectively).

Finally, our results were also robust with respect to the learning rule governing I → E connections. Although for simplicity, we have assumed the same plasticity rule governing all the plastic synapses (i.e. E → E, E → I and I → E), employing another plasticity rule for I → E connections [50] did not compromise our results (S5 Fig). We implemented this rule in S5 Fig, instead of our voltage-based rule before, and choose the parameters such that inhibitory plasticity ensures an average firing rate of 5 Hz in the excitatory population. Moreover, we used the denser connectivity of E → I connections corresponding to experimental data [4], instead of the default parameter. Note that given our parameters before, the connection frequency is now fully constrained by the experimental data (E → E and E → I connections according to [4] and I → E and I → I connections according to [51, 52]). To be more realistic, we also draw E → E connections from a normal distribution, similar to S2 Fig.

Similar results as before were also obtained in this case (S5 Fig). Initial orientation selective responses became sparser as a result of learning (S5A–S5D Fig), and feature-specific connectivity emerged in E → E connections (S5E–S5K Fig). Synaptic weights showed stable and convergent evolution under evoked state as well as spontaneous activity (S5L Fig), while change in the statistics of stimulus presentation was reflected in the statistics of feature-specific connectivity (S5M Fig). Note that neurons with similar preferred orientations are connected together with the strongest weights (S5H Fig), consistent with a recent experimental report [53]. This, however, does not render the network fragmenting into disconnected parts, as neurons with similar preferred orientations are connected together in a continuous manner. Thus, we can conclude that our main results hold in realistic balanced networks fully constrained with experimentally reported connectivity, and that they are robust to the main parameters of network dynamics and plasticity, including the I → E plasticity rule.

## Discussion

We analyzed biologically realistic models of sensory cortical networks, which are strongly driven by recurrent activity, and the recurrent connections of which undergo synaptic plasticity. We showed that functionally specific and stable connections can arise from an initially random structure. Specifically, we could demonstrate that Hebbian synaptic plasticity in balanced random networks can lead to the emergence of specific and bidirectional connectivity in orientation-selective networks. The initial response selectivity generated by such random networks guides the Hebbian learning process to enhance the connectivity between neurons with similar evoked responses.

If the initial selectivity is absent, or if it is very weak, specific connections, do not emerge, or remain very weak (Fig 9). Therefore, if the recurrent coupling is weak, or if the network is not inhibition-dominated, significant specific connectivity does not emerge. This is an immediate consequence of too weak initial tuning of output tuning curves. This sequence of events in the emergence of orientation selectivity and specific connectivity during learning is indeed fully consistent with the developmental processes, where neurons first show orientation selective responses, and only later functional subnetworks emerge [6].

The plastic mechanisms in our networks would also lead to the joint emergence of feature-specific neuronal responses and feature-specific synaptic connectivity, if the initial feature specificity was weak. Concretely, if not enough inhibition was provided at the beginning to ensure sharp and sparse output responses, a potentiation of inhibition in our networks lead to an enhancement of neuronal response selectivity during development. This was indeed accompanied by the simultaneous emergence of feature-specific synaptic connectivity in the network, demonstrating that self-regulation in a randomly connected balanced network via synaptic plasticity is capable of adjusting the network to work in the “right” regime, dominated by inhibition.

In fact, in absence of such a dynamic self-regulation, plasticity of excitatory connections can lead to a network, where only neurons with very similar orientation selectivity are connected together. A similar issue was recently raised by [3], where it is argued that Hebbian plasticity, naively applied, would drive networks to become overly specific, with only very few excitatory connections between neurons preferring different orientations. First, this scenario is inconsistent with the experimental results which show a heterogeneous functional connectivity in mouse visual cortex [54]. Second, an overrepresentation of connections between functionally similar neurons can lead to an instability of network dynamics, with pathological states of highly correlated activity (unpublished observation). None of these phenomena were observed in the simulations we report here, demonstrating that in plastic balanced networks self-regulatory mechanisms emerge that work against such consequences.

We found that the emergence of functionally specific connectivity was a robust process that always converged to a stable equilibrium in our simulations. However, the network was sensitive to the stimulus statistics, and an overrepresentation of specific stimulus orientations eventually increased the number of neurons that selectively responded to that orientation. Moreover, the synaptic weights in the equilibrium state after learning remained stable for a spontaneously active network, in absence of visual stimulation.

Our results suggested that synaptic plasticity can enhance orientation selectivity. Consistent with experimental studies [6, 35, 38], we observed an enhancement of orientation selectivity in tuning after learning, as indicated by the OSI of tuning curves. The overall responses of our networks became sparser, with fewer neurons in the network responding to each stimulus orientation. This is consistent with experimental results demonstrating a sparsification of neuronal activity in visual cortex after eye opening [55], and a general decrease in cortical activity between eye opening (postnatal day 13) and the peak of the critical period for rodent visual cortical plasticity (postnatal day 28) [56]. Also, consistent with our findings on the role of inhibition and stimulus statistics in refining both connectivity and function during development, a recent experimental study has found an enhancement of surround-suppression during normal vision for natural surround stimuli [57].

The importance of inhibition-dominance and inhibitory plasticity for our results brings up a question with regard to recent experimental studies, which argue that lateral inhibition is not a prerequisite for the emergence of orientation selectivity and its sharpening, and that the nonlinearity induced by the spiking threshold is enough [58–61]. First, in our networks, too, a nonlinearity induced by the spiking threshold was necessary for sharpening of tuning curves, as we have analyzed and discussed in a recent study [49]. In fact, if such a nonlinearity in the transfer function of single neurons was absent, no sharpening would appear, as a linear operation of the neuronal network would linearly transform the feedforward tuning to output tuning curves and map input cosine curves to output cosine curves [25, 49]. This nonlinearity, however, is not acting alone in our networks, as the interplay between recurrent (excitatory and inhibitory) inputs from the network and the nonlinearities of single cell transfer functions eventually determines the output orientation selectivity and its sharpening.

This notion is supported by recent experimental results in mouse visual cortex [62, 63], where broad inhibitory input underlies a developmental [63] and a contrast-dependent [62] sharpening of tuning curves. These results, along with recent optogenetic experiments on the contribution of inhibitory neurons to orientation selectivity [64, 65], provide direct evidence for the functional role of inhibition in this process. However, we cannot rule out the possibility of species-specific differences regarding the contribution of intracortical inhibition, especially between species with and without orientation maps (e.g. cats and macaque monkeys vs. mice and rats) [1, 66–68], although clear evidence for the contribution of untuned inhibition to orientation selectivity has also been provided for the former species [69, 70]. Further experimental studies are thus needed to resolve this issue, and to elucidate the relative importance of each mechanism.

The voltage triplet plasticity rule we used here was a non-linear variant with a symmetric component. It was constructed to account for experiments [31], which show that if both neurons fire at high rate (about 15 Hz), pre-before-post and post-before-pre both lead to LTP. In that case, two neurons with the same orientation preference tend to fire together at high rate, and therefore are bound to develop a bidirectional connection. An asymmetrical STDP rule, in contrast, would tend to remove bidirectional connections (see also [14]). Hence, the learning rule we used here is well suited to generate the connectivity pattern recently observed experimentally in visual cortex [5, 6, 71].

The form of the plasticity rule from E → E, E → I, and I → E in our work was assumed to be identical for the sake of simplicity. There are some recent data on inhibitory plasticity (e.g. [72, 73], see also [74] for a review), but the experimental evidence is rather confusing. The exact rule seems to be highly dependent on the protocol, the inhibitory neuron subtype, and the experimental preparation. Therefore, we chose the simplest possible approach and used the same rule for our main results. Specifically, our I → E plasticity rule was different from the plasticity rule employed in a previous study [50], which set out to reproduce excitatory and inhibitory co-tuning in auditory cortex [75], where inhibitory neurons are sharply tuned. Detailed balance resulted from this rule. In the visual cortex, although orientation selectivity of inhibitory neurons and the specificity of their wiring is controversial in the experimental literature [45, 76], mostly a non-specific connectivity of inhibitory neurons for both major subtypes (i.e. PV+ and SOM+ interneurons) has been reported (see e.g. [64]). We therefore confined our modeling to PV-expressing interneurons with broad tuning and non-specific connectivity to conform better to the currently available experimental data. Nevertheless, we were able to show that the same results can be obtained using the I → E plasticity rule of [50] (S5 Fig), or, in fact, even without plasticity of I → E connections (S1 Fig).

We assumed non-plastic feedforward connections in our study. The rationale behind this decision came from experimental results [6], which showed that orientation selectivity is already in place at eye opening, and only later on, presumably thanks to experience-dependent plasticity, there is a refinement of intra-cortical connections. Our present work puts a focus on modeling the second step of the development, i.e. what happens after eye-opening. We think that, even though the feedforward weights are still plastic during that time, they do not change orientation selectivity much anymore. Note that feedforward plasticity was studied in a previous model [6, 26], but the model did not have large recurrent and complex dynamics of excitation and inhibition. Here, instead, we focused on the cooperation of recurrent dynamics and recurrent plasticity, and we showed how functional networks can operate in balanced regimes of activity and develop feature-specific connectivity.

It has been shown that while Hebbian learning can extract the first principal component [77], including inhibition allows us to extract other components as well [78]. Inclusion of recurrent inhibition in realistic network models thus paves the road to study this phenomenon. It can also cast light on the formation of receptive fields: a non-linear spike-timing-dependent plasticity (STDP) rule has been recently shown [79] to reduce to a rule similar to the Bienenstock-Cooper-Munro (BCM) rule, which can form receptive fields [80]. The excitatory plasticity model (voltage-triplet rule, i.e. a non-linear variant of STDP) used in this paper allows to extract three point correlation structures in the inputs, as shown in [79]. If this rule is applied to patches of images that are whitened, receptive fields emerge that are Gabor filters, similar to orientation selectivity [26]. In contrast, using standard linear STDP learning, no Gabor filters can develop. Lateral inhibition can be used to make sure that neurons become selective to different orientations [26] so that the network can recover other components and represent all the different orientations.

A study using a pair-based STDP rule has shown that plasticity of recurrent excitatory connections may lead a group of neurons to take over the whole network, but including recurrent (nonplastic) inhibition prevents that and can lead to the emergence of maps [46]. The latter result is consistent with our network model here, where the balance of excitation and inhibition keeps the network in a dynamically stable regime, which in turn ensures a linear representation of the feedforward input [48, 49]. It is, however, difficult to replicate the same winner-take-all type of behavior reported in [46] in our networks for the following reasons. First, the activity in our networks is dominated by recurrent input to neurons. Hence, the networks show unstable activity in absence of recurrent inhibition, since the excitatory recurrent feedback is large. Second, the networks are dominated by recurrent plasticity. As a result, when recurrent inhibition is not present, the output selectivity of responses is very weak and the output selectivity cannot be enhanced by feedforward plasticity. Because the activity is very weakly selective, the selective component of the responses cannot dominate in a winner-take-all type of dynamics. The network response, instead, ends up in a pathological state of nonselective activity, where all neurons are active (not shown).

This was not the case, however, for smaller, feedforward-driven networks where recurrent dynamics did not lead to runaway excitation in absence of inhibition, and where the feedforward input was dominant such that the receptive fields of neurons were mainly determined by it (networks developed in [6, 26]). The same winner-take-all behavior as reported in [46] was observed in absence of inhibition, where all neurons developed the same selectivity. It will be interesting to see in the future how including feedforward plasticity can change the behavior of our network model in this respect. This might be particularly pertinent to experimental findings on brain plasticity after lesion, and useful for understanding the mechanisms of brain repair in certain brain diseases.

In conclusion, our study demonstrates how functional balanced networks with realistic recurrent dynamics can work in tandem with Hebbian synaptic plasticity to understand and explain findings recently reported in experimental studies. It sheds light on the process of refinement of network responses during development, and elucidates the conditions for the emergence of feature-specific connectivity in the network. Our results underline the significant role of inhibition for network function [81], both for the emergence of feature selectivity and feature-specific connectivity. This work in turn, triggers another important question: Which functional properties result from feature-specific connectivity in the network [82]? Further theoretical and experimental studies are definitely needed to address these questions, but our present study indicates some promising directions to further explore large-scale neuronal networks with realistic recurrent dynamics and synaptic plasticity.

## Materials and Methods

### Network model

The technical details of network model are described elsewhere [25]. Briefly, the model consists of a recurrent network of *N* = 500 leaky integrate-and-fire (LIF) neurons, of which *f* = 80% are excitatory and 20% are inhibitory [83]. The sub-threshold dynamics of the membrane potential *u*_*k*_(*t*) of neuron *k* is given by the leaky-integrator equation

$$\begin{array}{c} \tau {\dot{u}}_{k}\left(t\right)+u_{k}\left(t\right)=RI_{k}\left(t\right). \end{array}$$ (1)

The current *I*_*k*_(*t*) represents the total input to neuron *k*, the (leaky) integration of which is governed by the leak resistance *R*, and the membrane time constant *τ* = 20 ms. When the voltage reaches the threshold at *u*_th_ = 20 mV, a spike is generated and transmitted to all post-synaptic neurons, and the membrane potential is reset to the resting potential at *u*_0_ = 0 mV.

Each excitatory neuron projects to a randomly sampled population comprising *ϵ*_exc_ = 30% of all neurons in the network. Inhibitory neurons project to all neurons in the network (*ϵ*_inh_ = 100%) [51, 52]. In addition, inhibitory post-synaptic potentials have a *g* = 8 times larger amplitude than excitatory ones [9, 25]. This choice of parameters is motivated by the dense connectivity of inhibitory neurons reported in different areas of cortex [4, 51, 84]. Post-synaptic currents are modeled as *δ*-functions, where the total charge *Q* is delivered instantaneously to the post-synaptic neuron after the arrival of a spike, without a synaptic delay. Synaptic coupling strength is measured in terms of the amplitude of the resulting post-synaptic potential (PSP), *J* = *QR*/*τ*. Unless stated otherwise, the amplitude of excitatory connections in the local network is *J*_exc_ = 0.5 mV, and that of inhibitory connections *J*_inh_ = −*gJ*_exc_ = −4 mV.

Upon presentation of the visual stimulus, an elongated bar with some fixed orientation, feedforward input is driving cortical neurons. We lump all feedforward synapses to a given neuron into one single synapse, and model the feedforward input as a single channel with an untuned and a tuned component. The untuned baseline firing rate of this feedforward input is *s*_*b*_ = 2 kHz, and the amplitude of the input synapses is *J*_ffw_ = 1 mV. The tuned component of the input is modulated depending on the orientation of the stimulus, *θ*, and the preferred orientation (PO) of the neuron, θ*, according to a cosine function

$$\begin{array}{c} s\left(\theta ,\theta^{*}\right)=s_{b}[1+\mu \cos(2\left(\theta -\theta^{*}\right))]. \end{array}$$ (2)

The parameter *μ* is the relative modulation amplitude of the input tuning curve, which is set to *μ*_exc_ = 20% for excitatory neurons, and *μ*_inh_ = 2% for inhibitory neurons, consistent with weaker tuning of inhibitory neurons reported in the cortex [4, 44, 45].

In our simulations, the feedforward input is represented by a stationary Poisson process of rate *s*. To obtain individual tuning curves, as shown in Fig 2, the stimulation of the network is repeated with 8 different orientations, 0°, 22.5°, 45°, …, 157.5°. Selectivity of responses, including individual tuning curves, is quantified by a global selectivity index, OSI = 1−circular variance [25, 36].

The implementation of the LIF model is based on a numerical method known as “exact integration” [85, 86]. Numerical integration of network dynamics was performed using a time step of *dt* = 1 ms. We repeated some of the simulations with a smaller time step (*dt* = 0.1 ms) to verify the accuracy of our results.

The simulation code will be published on the freely-available repository ModelDB (http://senselab.med.yale.edu/modeldb/) after publication.

### Plasticity model

The plasticity model is explained in detail elsewhere [6, 26]. Here, it applies only to recurrent weights, and describes changes in the synaptic amplitude of synapse *i* (*w*_*i*_) by the equation

$$\begin{array}{c} \frac{d}{dt}w_{i}=-A_{\text{LTD}}\left(\bar{\bar{u}}\right)\;X_{i}\left(t\right)\;{\left[{\bar{u}}_{-}\left(t\right)-\theta_{-}\right]}_{+}+A_{\text{LTP}}\;{\bar{x}}_{i}\left(t\right)\;{\left[u\left(t\right)-\theta_{+}\right]}_{+}\;{\left[{\bar{u}}_{+}\left(t\right)-\theta_{-}\right]}_{+}, \end{array}$$ (3)

combined with hard bounds *w*_min_ ≤ *w*_*i*_ ≤ *w*_max_. *w*_min_ and *w*_max_ are 0 mV and 2 mV for excitatory synapses, and −5 mV and 0 mV for inhibitory synapses, respectively.

The quantity ${\bar{u}}_{-}(t)$ is a low-pass filtered version of the postsynaptic membrane potential *u*(*t*), assuming an exponential kernel with time constant *τ*_−_:

$$\begin{array}{c} \tau_{-}\frac{d}{dt}{\bar{u}}_{-}\left(t\right)=-{\bar{u}}_{-}\left(t\right)+u\left(t\right). \end{array}$$ (4)

The brackets [.]_+_ denote half-wave rectification ([*x*]_+_ = *x* for *x* > 0, and [*x*]_+_ = 0 otherwise) and reflect experimental findings showing that postsynaptic depolarization induces a depression of the synapse only if it exceeds a certain threshold value *θ*_−_ [29]. The presynaptic spike train is represented by a sequence of impulses at times $t_{i}^{n}$, $X_{i}(t)=\sum_{n}\delta (t-t_{i}^{n})$, where *i* is the index of the synapse and *n* is an index that counts the spike. $A_{\text{LTD}}(\bar{\bar{u}})$ is an amplitude parameter that is under the control of a homeostatic processes [87]. Here it depends on $\bar{\bar{u}}$, which is the mean depolarization of the postsynaptic neuron, averaged over a time window of size 0.1 s: $A_{\text{LTD}}(\bar{\bar{u}})=A_{\text{LTD}}\;\frac{{\bar{\bar{u}}}^{2}}{u_{ref}^{2}}$ where $u_{ref}^{2}$ is a reference value. The variable $\bar{\bar{u}}$ is used to control the homeostasis, and therefore the window is longer than all the other time constants. *u*_ref_ defines the target depolarization that the homeostasis is reaching, whereas *A*_LTD_ is the amplitude for depression of the rule. See [26] for more details.

For the LTP part, we assume that each presynaptic spike arriving at the synapse *w*_*i*_ increases a synapse-specific trace ${\bar{x}}_{i}(t)$ of some biophysical quantity by some fixed value. The trace ${\bar{x}}_{i}(t)$ decays exponentially with a time constant *τ*_*x*_ in the absence of presynaptic spikes, as described in previous work [43, 88]. The temporal evolution of ${\bar{x}}_{i}(t)$ is described by a linear differential equation

$$\begin{array}{c} \tau_{x}\frac{d}{dt}{\bar{x}}_{i}\left(t\right)=-{\bar{x}}_{i}\left(t\right)+X_{i}\left(t\right), \end{array}$$ (5)

where *X*_*i*_ is the input spike train defined above. *A*_LTP_ is a free parameter inferred from experimental data, and ${\bar{u}}_{+}(t)$ is another lowpass filtered version of *u*(*t*) similar to ${\bar{u}}_{-}(t)$, but with a somewhat shorter time constant *τ*_+_. Thus positive weight changes can occur if the momentary voltage *u*(*t*) surpasses a threshold *θ*_+_ and, at the same time, the average value ${\bar{u}}_{+}(t)$ is above *θ*_−_.

The two time constants *τ*_+_ and *τ*_−_ were fitted to several experimental data (see [26]). Without the filtering, it is neither possible to reproduce the frequency dependency demonstrated in [31], nor the classical spike-timing dependent plasticity learning window. The voltage is very fast during a spike and therefore needs to be filtered to detect the pre-post coincidence. This rule shows a nice trade-off between the number of parameters and the robustness at describing the experimental phenomenology [34].

The values of the plasticity parameters in our simulations are: *τ*_−_ = 10 ms, *τ*_+_ = 7 ms, *τ*_*x*_ = 15 ms, *A*_LTD_ = 14 × 10^−5^, *A*_LTP_ = 8 × 10^−5^, *θ*_−_ = −20 mV, *θ*_+_ = 7.5 mV, $u_{ref}^{2}=70\;{\text{mV}}^{2}$. Structural plasticity is not allowed: only synapses that were initially established undergo potentiation and depression. Feedforward weights are not plastic. For a justification of network and plasticity parameters, see [25, 26], respectively. All the parameters are listed in Table 1.

**Table 1:** Table of parameters. Default parameters are given, and only the exceptions are noted for figures with parameters different from the default values.

Parameters of	Default values	Fig 8	Fig 9	Fig 10	S1 Fig	S2 Fig	S3 Fig	S4 Fig	S5 Fig
**Single Neurons**									
membrane time constant	*τ*_m_ = 20 ms								
resting potential	*V*_rest_ = 0 mV								
threshold voltage	*V*_th_ = 20 mV								
refractory period	*t*_ref_ = 0 ms								
**Network Simulations**									
# neurons	*N* = 500								
fraction of Exc neurons	*f* = 80%								
fraction of Inh neurons	1−*f* = 20%								
Exc to Exc conn. prob.	*ϵ*_E → E_ = 30%								
Exc to Inh conn. prob.	*ϵ*_E → I_ = 30%				80%				80%
Inh to Exc conn. prob.	*ϵ*_I → E_ = 100%								
Inh to Inh conn. prob.	*ϵ*_I → I_ = 100%								
recurrent EPSP (mV)	*J*_exc_ = 0.5				0.1	𝓝(0.5, 0.5)			𝓝(0.1, 0.1)
recurrent IPSP: *J*_inh_ = −*gJ*_exc_	*g* = 8	4	(1, …, 8)		4				2
feedforward EPSP (mV)	*J*_ffw_ = 1								
feedforward baseline rate	*ν*_ffw_ = 2 kHz								
modulation ratio of Exc input	*μ*_exc_ = 20%								
modulation ratio of Inh input	*μ*_inh_ = 2%								
**Synaptic Plasticity**									
*θ*_+_	7.5 mV			±10%					
*θ*_−_	−20 mV			±10%					
*A*_LTP_	8 × 10^−5^			±10%			+20% for Exc	+20% for Inh	
*A*_LTD_	14 × 10^−5^			±10%					
*τ*_−_	10 ms								
*τ*_+_	7 ms								
*τ*_x_	15 ms								
plasticity of I → E	[26]								[50]

To quantify the amount of bidirectional connectivity emerging as a result of plasticity (as in e.g. Fig 3) we used a weighted bidirectionality index (WBI). As we did not allow for structural plasticity in our networks, we quantified this by computing the product of bidirectional weights between each pair of neurons (*w*_*ij*_ *w*_*ji*_ for neurons *i* and *j*). If only one neuron is connected to the other, and the bidirectional connection is completely missing (*w*_*ij*_ = 0 or *w*_*ji*_ = 0), the product is zero. To obtain WBI of a network, we average over all neuronal pairs in the network. A completely unidirectionally connected network would therefore return a WBI of zero. For networks with bidirectional connections, WBI would be larger than zero. For a specific pair of neurons with a similar sum of weights, *w*_*ij*_ + *w*_*ji*_ = constant, WBI would be highest if weights are fully bidirectional, i.e. *w*_*ij*_ = *w*_*ji*_.

To account for the fact that some bidirectional connectivity exists by chance in random networks, we shuffle the elements of weight matrix for each network and compute its WBI as WBI_random_. The normalized WBI, WBI_norm_, is then obtained by dividing the WBI of the actual weight matrix of the network by its shuffled WBI: WBI_norm_ = WBI/WBI_random_.

## Supporting Information

S1 Fig Emergence of specific connectivity in a network with a different initial connectivity. Results are illustrated in the same fashion as in Fig 8, for a network with the following parameters of connectivity: probability of an I → E connection = 80%, *J* = 0.1, *g* = 4. I → E weights are not plastic. Other parameters are the same as the default values. The learning phase is organized in 20 batches.
(TIF)

S2 Fig Effect of the initial distribution of excitatory weights. Same as Fig 6 for the same network, but with a different initial weight matrix. Instead of excitatory weights being either 0 or *J*_exc_, as in Fig 6, here the amplitudes of non-zero initial connections are drawn from a Gaussian distribution with mean *J*_exc_ and standard deviation *J*_exc_. Rarely occurring negative values are set to zero.
(TIF)

S3 FigEffect of an altered potentiation rate of excitatory connections.The default network with the same parameters as the network in Fig 1, except for a potentiation rate of excitatory (to both excitatory and inhibitory) synapses increased by 20%: $A_{\text{LTP}}^{\text{exc}}=9.6\times 10^{-5}$. Panels and conventions are the same as in Fig 8.(TIF)

S4 FigEffect of an altered potentiation rate of inhibitory connections.The default network with the same parameters as the network in Fig 1, except for a potentiation rate of inhibitory (to excitatory) synapses increased by 20%: $A_{\text{LTP}}^{\text{inh}}=9.6\times 10^{-5}$. Panels and conventions are the same as in Fig 8.(TIF)

S5 FigEmergence and evolution of specific connectivity in a network with denser connectivity of E → I and a different plasticity rule for I → E connections.Probability of an I → E connection is 80% [4], *J*_exc_ = 0.1, *g* = 2. Similar to to S2 Fig, the amplitudes of non-zero initial E → E connections are drawn from a Gaussian distribution with mean *J*_exc_ and standard deviation *J*_exc_. I → E connections are plastic according to the plasticity rule described in [50]. Briefly, the synaptic weight *w*_ij_ from a pre-synaptic inhibitory neuron *j* to a post-synaptic excitatory neuron *i* is updated at each time step according to the following rule: $w_{\text{ij}}\leftarrow w_{\text{ij}}+\eta ({\bar{x}}_{i}-\alpha )$ for pre-synaptic spikes, and $w_{\text{ij}}\to w_{\text{ij}}+\eta {\bar{x}}_{j}$ for post-synaptic spikes [50]. Here, ${\bar{x}}_{j}$ and ${\bar{x}}_{i}$ are traces obtained by low-pass filtering (similar to Eq 5 with the same time constant *τ*_*x*_) spikes emitted in the pre- and post-synaptic neurons *j* and *i*, respectively. *η* = 0.1 is a learning rate and *α* = 0.01 is a depression factor. The parameters are chosen to ensure an output post-synaptic firing rate of 5 Hz. Other parameters are the same as the default values. The learning phase is organized in 40 batches. For the spontaneous activity, the network is stimulated with an untuned input with *s*_*b*_. Panels and conventions are the same as in Figs 6–8 of the main text with the following correspondence: panels **(A–K)** correspond to Fig 8, A–k; panel (**L**) corresponds to Fig 6A; and panel (**M**) corresponds to Fig 7A.(TIF)
