# Supplementary figures and images for: Emergence of Functional Specificity in Balanced Networks with Synaptic Plasticity
Source: PLoS Comput Biol. 2015 Jun 19;11(6):e1004307. doi: 10.1371/journal.pcbi.1004307 (PMC4474917; doi:10.1371/journal.pcbi.1004307)

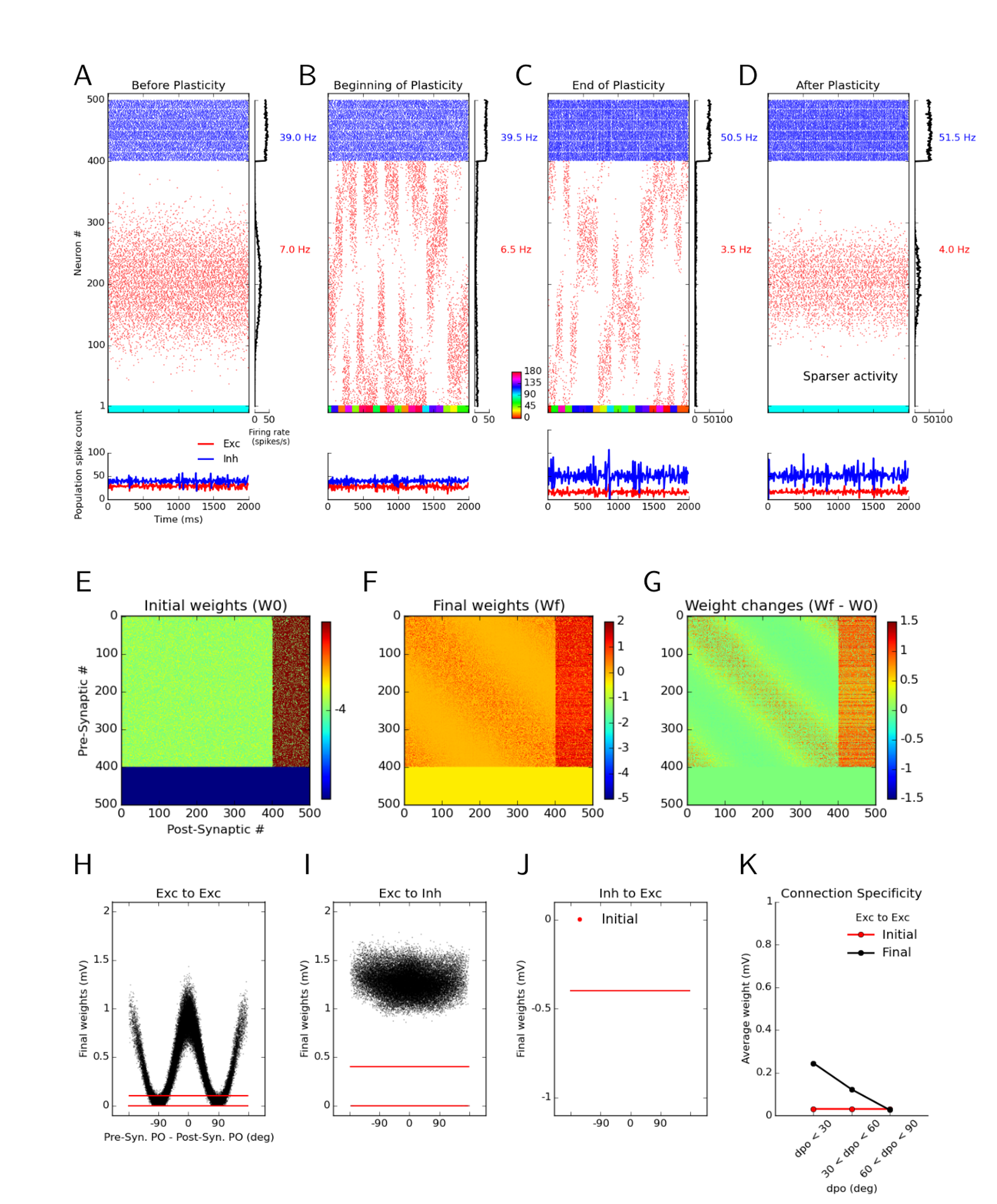

Supplement: S1 Fig — Results are illustrated in the same fashion as in Fig 8, for a network with the following parameters of connectivity: probability of an I → E connection = 80%, J = 0.1, g = 4. I → E weights are not plastic. Other parameters are the same as the default values. The learning phase is organized in 20 batches. (TIF) [file pcbi.1004307.s001.tif]

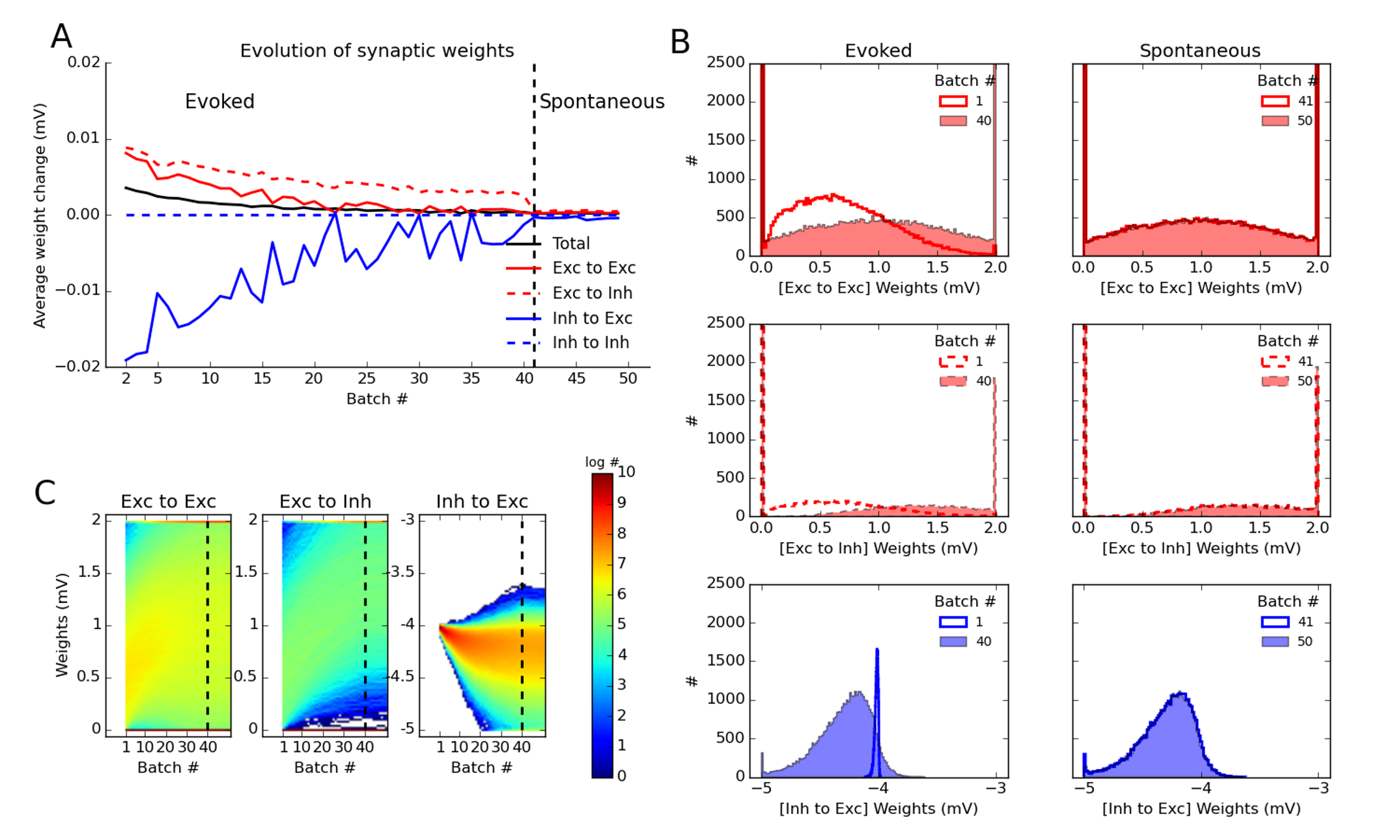

Supplement: S2 Fig — Same as Fig 6 for the same network, but with a different initial weight matrix. Instead of excitatory weights being either 0 or J exc, as in Fig 6, here the amplitudes of non-zero initial connections are drawn from a Gaussian distribution with mean J exc and standard deviation J exc. Rarely occurring negative values are set to zero. (TIF) [file pcbi.1004307.s002.tif]

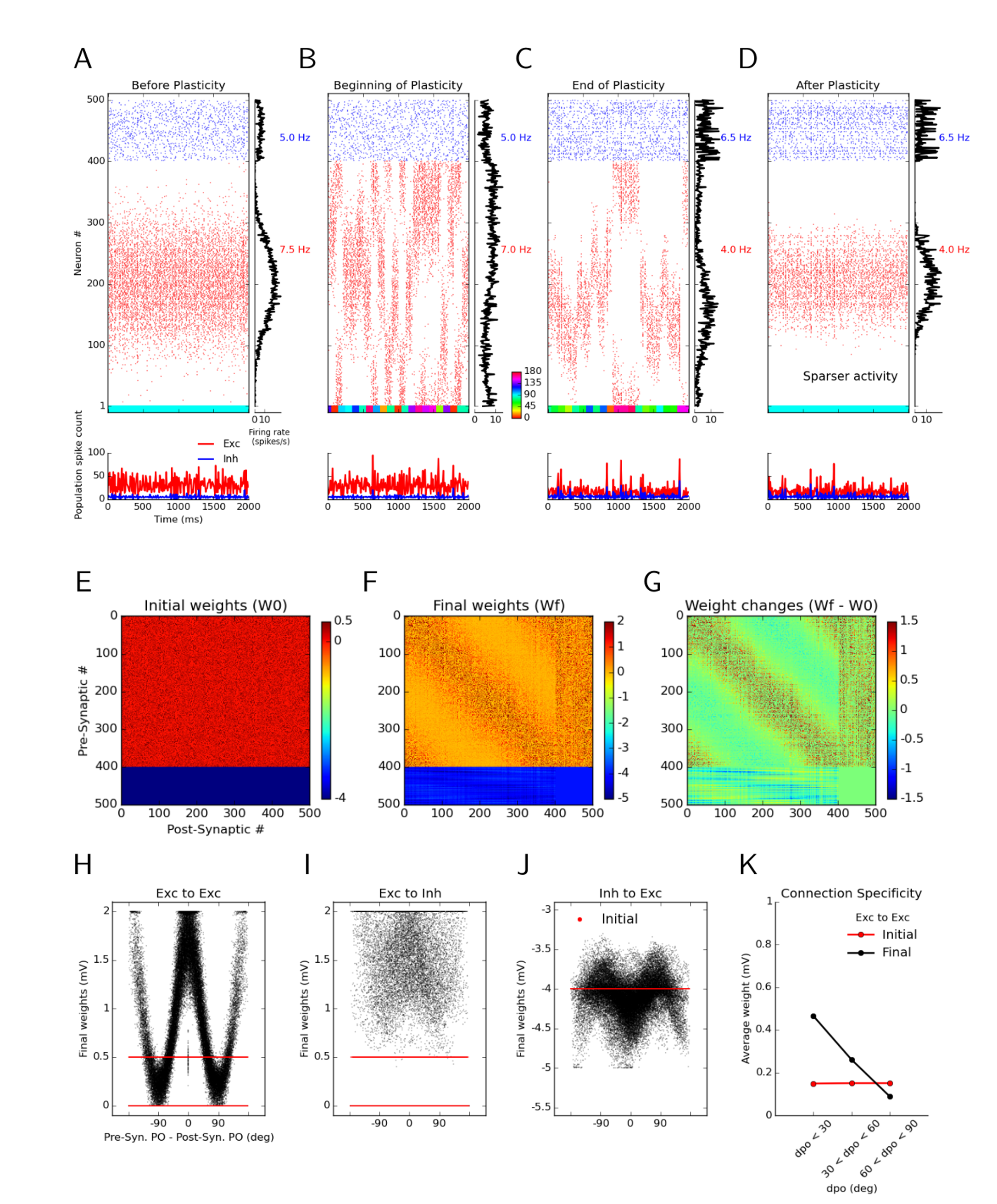

Supplement: S3 Fig — The default network with the same parameters as the network in Fig 1, except for a potentiation rate of excitatory (to both excitatory and inhibitory) synapses increased by 20%: ALTPexc=9.6×10^−5^. Panels and conventions are the same as in Fig 8. (TIF) [file pcbi.1004307.s003.tif]

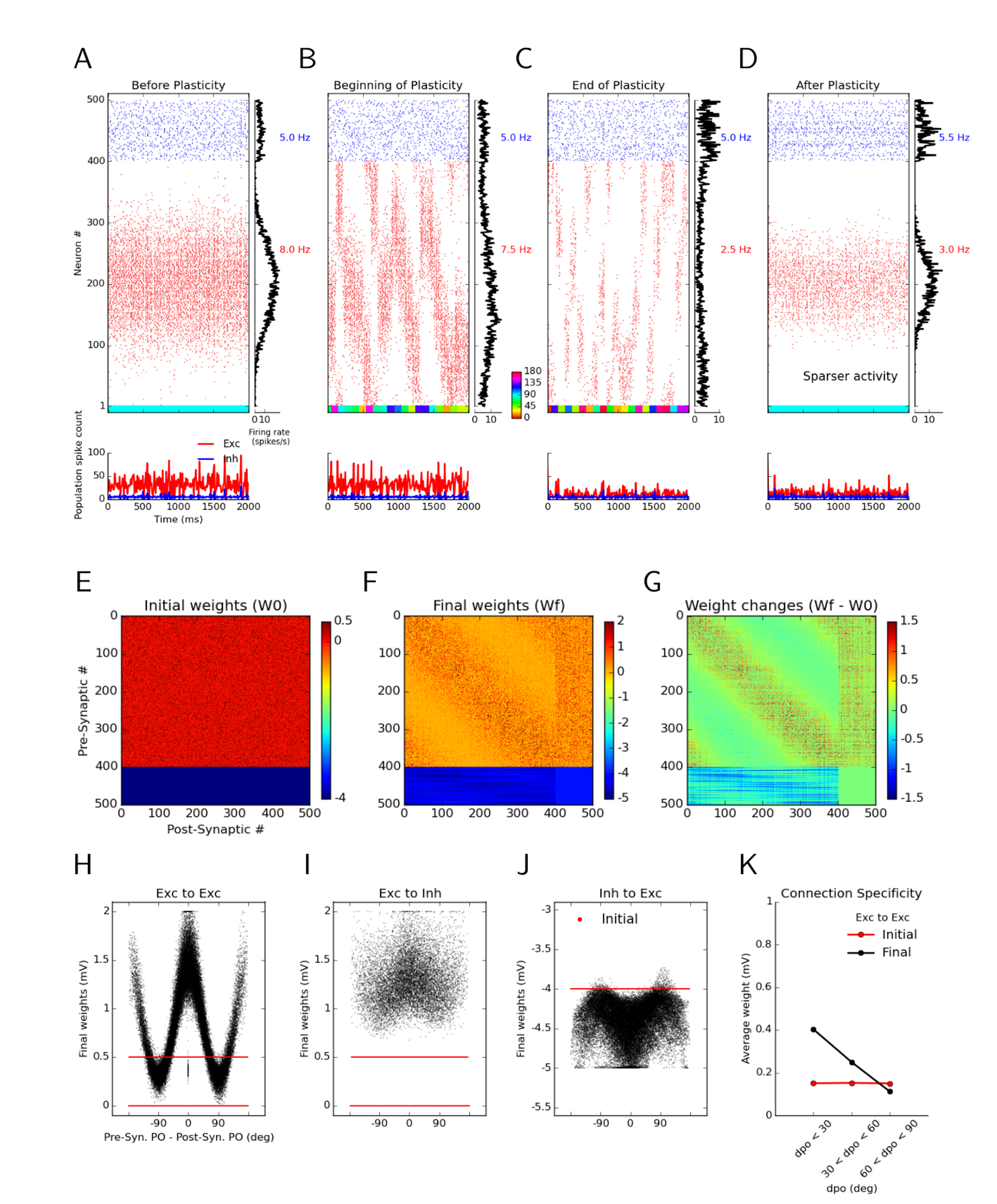

Supplement: S4 Fig — The default network with the same parameters as the network in Fig 1, except for a potentiation rate of inhibitory (to excitatory) synapses increased by 20%: ALTPinh=9.6×10^−5^. Panels and conventions are the same as in Fig 8. (TIF) [file pcbi.1004307.s004.tif]

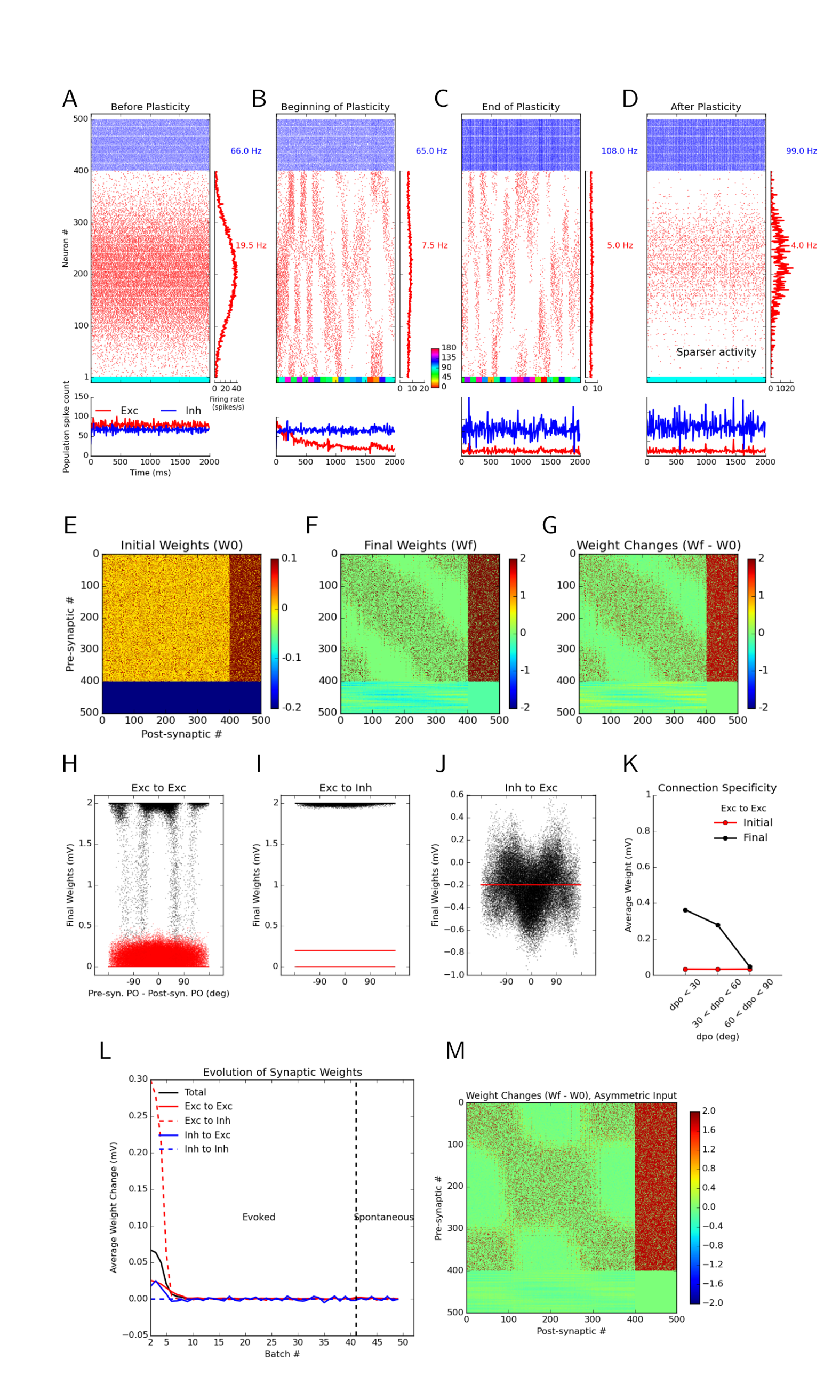

Supplement: S5 Fig — Probability of an I → E connection is 80% [4], J exc = 0.1, g = 2. Similar to to S2 Fig, the amplitudes of non-zero initial E → E connections are drawn from a Gaussian distribution with mean J exc and standard deviation J exc. I → E connections are plastic according to the plasticity rule described in [50]. Briefly, the synaptic weight w ij from a pre-synaptic inhibitory neuron j to a post-synaptic excitatory neuron i is updated at each time step according to the following rule: wij←wij+η(x‾i−α) for pre-synaptic spikes, and wij→wij+ηx‾j for post-synaptic spikes [50]. Here, x‾j and x‾i are traces obtained by low-pass filtering (similar to Eq 5 with the same time constant τ x) spikes emitted in the pre- and post-synaptic neurons j and i, respectively. η = 0.1 is a learning rate and α = 0.01 is a depression factor. The parameters are chosen to ensure an output post-synaptic firing rate of 5 Hz. Other parameters are the same as the default values. The learning phase is organized in 40 batches. For the spontaneous activity, the network is stimulated with an untuned input with s b. Panels and conventions are the same as in Figs 6–8 of the main text with the following correspondence: panels (A–K) correspond to Fig 8, A–k; panel (L) corresponds to Fig 6A; and panel (M) corresponds to Fig 7A. (TIF) [file pcbi.1004307.s005.tif]
